# Complete Genome Sequence of Pseudomonas aeruginosa Mucoid Strain FRD1, Isolated from a Cystic Fibrosis Patient

**DOI:** 10.1128/genomeA.00153-15

**Published:** 2015-03-19

**Authors:** Laura A. Silo-Suh, Sang-Jin Suh, Dennis E. Ohman, Daniel J. Wozniak, Julia W. Pridgeon

**Affiliations:** a Department of Basic Medical Sciences, Mercer University School of Medicine, Macon, Georgia, USA; b Department of Biological Sciences, Auburn University, Auburn, Alabama, USA; c Department of Microbiology and Immunology and Virginia Commonwealth University Medical Center, Richmond, Virginia, USA; d Hunter Holmes McGuire VA Medical Center, Richmond, Virginia, USA; e Center for Microbial Interface Biology, The Ohio State University, Columbus, Ohio, USA; f Aquatic Animal Health Research Unit, USDA-ARS, Auburn, Alabama, USA

## Abstract

We announce here the complete genome sequence of the Pseudomonas aeruginosa mucoid strain FRD1, isolated from the sputum of a cystic fibrosis patient. The complete genome of P. aeruginosa FRD1 is 6,712,339 bp. This genome will allow comparative genomics to be used to identify genes associated with virulence, especially those involved in chronic pulmonary infections.

## GENOME ANNOUNCEMENT

The Gram-negative bacterium Pseudomonas aeruginosa is the major etiological agent responsible for pulmonary infections in cystic fibrosis (CF) patients leading to morbidity and mortality. CF patients are frequently colonized by environment-borne P. aeruginosa, which initially causes intermittent infections (1). Chronic infections are associated with significant genetic changes in the bacterium that appear to facilitate the persistence of the pathogen in this niche (2, 3). One such change is the acquisition of the mucoid phenotype caused by mutations in the *mucA* gene leading to overproduction of the exopolysaccharide alginate (4, 5). Alginate is a major component of the P. aeruginosa biofilm matrix responsible for increased tolerance to antibiotics (6). A mucoid strain of P. aeruginosa, designated FRD1, was isolated from a sputum sample from a chronically infected CF patient in 1979 by Barbara Iglewski (Portland, OR) and first reported in 1981 as a strain that can be readily manipulated genetically (7). This isolate is arguably the best characterized CF P. aeruginosa isolate and has provided the basis for much of the information assembled on alginate biosynthesis and regulation in P. aeruginosa (8–11). The complete genome sequence of P. aeruginosa FRD1 was determined in this study in order to augment and facilitate a further investigation of the microevolution process of P. aeruginosa adaptation in the CF lung.

Genomic DNA was isolated from an overnight broth culture of P. aeruginosa FRD1 using the Wizard genomic DNA purification kit (Promega) and sequenced using the Pacific Biosciences RS II platform. HGAP3 was used to *de novo* assemble a total of 117,765 sequence reads, with an average length of 4,559 bp. The whole genome of P. aeruginosa FRD1 is 6,712,339 bp, with a G+C content of 66.1% and 6,439 predicted open reading frames. The contigs of the *de novo*-assembled genome of P. aeruginosa FRD1 share 99% identity with those of the genome of P. aeruginosa strain PA38182 (GenBank accession no. HG530068), a major epidemic strain throughout Europe (12). The annotation of the genome by Manatee detected the following breakdown within subsystems: 91 open reading frames in amino acid biosynthesis, 82 in fatty acid and phospholipid metabolism, 58 in central intermediary metabolism, 320 in energy metabolism, 1,030 in transport and binding proteins, 117 in DNA metabolism, 90 in transcription, and 273 in cell envelope.

### Nucleotide sequence accession number.

The complete genome sequence of P. aeruginosa FRD1 was deposited in GenBank under the accession no. CP010555. The version described in this paper is the first version.

## References

[1] Burns JL, Gibson RL, McNamara S, Yim D, Emerson J, Rosenfeld M, Hiatt P, McCoy K, Castile R, Smith AL, Ramsey BW 2001 Longitudinal assessment of *Pseudomonas aeruginosa* in young children with cystic fibrosis. J Infect Dis 183:444–452. doi:10.1086/318075. 11133376

[2] Nguyen D, Singh PK 2006 Evolving stealth: genetic adaptation of *Pseudomonas aeruginosa* during cystic fibrosis infections. Proc Natl Acad Sci U S A 103:8305–8306. doi:10.1073/pnas.0602526103. 16717189 PMC1482488

[3] Hoboth C, Hoffmann R, Eichner A, Henke C, Schmoldt S, Imhof A, Heesemann J, Hogardt M 2009 Dynamics of adaptive microevolution of hypermutable *Pseudomonas aeruginosa* during chronic pulmonary infection in patients with cystic fibrosis. J Infect Dis 200:118–130. doi:10.1086/599360. 19459782

[4] Doggett RG 1969 Incidence of mucoid *Pseudomonas aeruginosa* from clinical sources. Appl Microbiol 18:936–937. 4984207 10.1128/am.18.5.936-937.1969 PMC378118

[5] Martin DW, Schurr MJ, Mudd MH, Govan JR, Holloway BW, Deretic V 1993 Mechanism of conversion to mucoidy in *Pseudomonas aeruginosa* infecting cystic fibrosis patients. Proc Natl Acad Sci U S A 90:8377–8381. doi:10.1073/pnas.90.18.8377. 8378309 PMC47359

[6] Hentzer M, Teitzel GM, Balzer GJ, Heydorn A, Molin S, Givskov M, Parsek MR 2001 Alginate overproduction affects *Pseudomonas aeruginosa* biofilm structure and function. J Bacteriol 183:5395–5401. doi:10.1128/JB.183.18.5395-5401.2001. 11514525 PMC95424

[7] Ohman DE, Chakrabarty AM 1981 Genetic mapping of chromosomal determinants for the production of the exopolysaccharide alginate in a *Pseudomonas aeruginosa* cystic fibrosis isolate. Infect Immun 33:142–148. 6790439 10.1128/iai.33.1.142-148.1981 PMC350668

[8] Chitnis CE, Ohman DE 1990 Cloning of *Pseudomonas aeruginosa algG*, which controls alginate structure. J Bacteriol 172:2894–2900. 2160929 10.1128/jb.172.6.2894-2900.1990 PMC209086

[9] Franklin MJ, Ohman DE 1993 Identification of *algF* in the alginate biosynthetic gene cluster of *Pseudomonas aeruginosa* which is required for alginate acetylation. J Bacteriol 175:5057–5065. 8394313 10.1128/jb.175.16.5057-5065.1993 PMC204972

[10] Wozniak DJ, Ohman DE 1994 Transcriptional analysis of the *Pseudomonas aeruginosa* genes *algR*, *algB*, and *algD* reveals a hierarchy of alginate gene expression which is modulated by *algT*. J Bacteriol 176:6007–6014. 7928961 10.1128/jb.176.19.6007-6014.1994 PMC196818

[11] Jain S, Ohman DE 1998 Deletion of *algK* in mucoid *Pseudomonas aeruginosa* blocks alginate polymer formation and results in uronic acid secretion. J Bacteriol 180:634–641. 9457868 10.1128/jb.180.3.634-641.1998 PMC106932

[12] Witney AA, Gould KA, Pope CF, Bolt F, Stoker NG, Cubbon MD, Bradley CR, Fraise A, Breathnach AS, Butcher PD, Planche TD, Hinds J 2014 Genome sequencing and characterization of an extensively drug-resistant sequence type 111 serotype O12 hospital outbreak strain of *Pseudomonas aeruginosa*. Clin Microbiol Infect 20:O609–O618. doi:10.1111/1469-0691.12528. 24422878

